# Cerebrovascular phenotype analysis in *Gucy1a3* loss-of-function mice: insights into moyamoya disease susceptibility

**DOI:** 10.3389/fneur.2026.1669177

**Published:** 2026-04-13

**Authors:** Pingkai Wang, Tian Wang, Jipeng Yu, Jianli Li, Xiaozuo Lin, Yinan Zeng, Xiaohan Zhang, Shanghua Su, Man Luo

**Affiliations:** Department of Neurology, First Affiliated Hospital of Guangxi Medical University, Nanning, China

**Keywords:** cerebrovascular phenotype, *Gucy1a3* loss-of-function mice, magnetic resonance angiography (MRA), moyamoya disease, vasoconstrictive remodeling

## Abstract

**Background:**

Moyamoya disease (MMD) is characterized by progressive stenosis of the internal carotid artery and compensatory moyamoya angiogenesis. Although multiple studies have identified *GUCY1A3* (encoding the α1 subunit of soluble guanylate cyclase) as a susceptibility gene for MMD, genome‑wide association studies (GWAS) have not yet established it as a significant locus. To address this discrepancy, this study used *Gucy1a3* loss‑of‑function mice (*Gucy1a3*^−/−^) to investigate the potential causal relationship between *GUCY1A3* loss‑of‑function and the cerebrovascular phenotype in MMD.

**Methods:**

Intracranial arterial anatomy was assessed using 7.0T high‑resolution magnetic resonance angiography (MRA) and cerebral vascular casting. Vasoconstrictive remodeling was evaluated by calculating diameter ratios – specifically, internal carotid artery (ICA)/basilar artery (BA) and middle cerebral artery (MCA)/BA ratios. Histopathological evaluation was performed using hematoxylin and eosin (H&E) staining, elastic van Gieson (EVG) staining, and α‑smooth muscle actin (α‑SMA) immunohistochemistry to assess intracerebral macrovascular pathology. Cortical microvascular density and caliber were quantified using CD31 immunohistochemistry, while leptomeningeal vascular architecture was systematically analyzed using vascular casting, followed by skeletonization and topological analysis.

**Results:**

*Gucy1a3*^−/−^ developed normally, with no significant differences in intracranial vascular anatomy, diameter ratios, or large‑vessel histopathology compared to wild‑type mice (Wt). However, the leptomeningeal vascular network in *Gucy1a3*^−/−^ exhibited significant simplification, characterized by reduced vascular branching (365.90 ± 14.92 versus 330.00 ± 9.72, *p* = 0.0003) and density (total junctions, 174.10 ± 10.43 versus 155.80 ± 11.39, *p* = 0.0101; Vascular area%, 29.57 ± 4.40 versus 20.75 ± 2.01, *p* = 0.0021). In addition, cortical microvessel density (vascular density/ mm2, 515.70 ± 15.53 versus 351.20 ± 80.69, *p* = 0.0022; area%, 0.83 ± 0.20 versus 0.44 ± 0.18, *p* = 0.0152) and average diameter (3.5mm versus 2.5mm) were significantly reduced in *Gucy1a3*^−/−^.

**Conclusion:**

Under early adult (8–12 weeks) conditions, *GUCY1A3* deficiency did not produce detectable large‑artery stenosis typical of MMD. Instead, it induced a unique cerebrovascular phenotype marked by “rarefaction” of both cortical microvessels and leptomeningeal networks. Whether large‑vessel pathology develops in aged mice remains to be determined. These findings suggest that *GUCY1A3* loss‑of‑function may contribute to MMD susceptibility primarily by compromising small‑vessel integrity, necessitating additional triggers for full disease manifestation.

## 1 Introduction

Moyamoya disease (MMD) represents a rare, chronic progressive cerebrovascular disorder characterized by progressive stenosis or occlusion of the terminal internal carotid arteries and their proximal branches, accompanied by the compensatory development of an abnormal collateral vascular network at the base of the brain (designated moyamoya vessels) (1). Pathological hallmarks of MMD include intimal hyperplasia with smooth muscle cell proliferation, medial layer thinning, and luminal thrombus formation, which together promote progressive arterial stenosis and attendant stroke risks (2, 3). The disease exhibits distinct epidemiological features: a bimodal age distribution, with peaks in childhood (5–9 years) and middle adulthood (40–49 years), a significantly higher incidence in East Asian populations compared with other global regions, with 12.1% familial aggregation and a 34-fold increased risk in first-degree relatives, underscoring the disease’s heritable basis (4, 5).

Despite extensive research, the precise etiology of MMD remains elusive, with hypotheses implicating genetic predisposition, autoimmune processes, and environmental triggers (6–8). Among candidate susceptibility genes, the guanylate cyclase soluble subunit alpha-1(*GUCY1A3*) gene, mapped to chromosome 4q32.1, encodes the α1 subunit of soluble guanylate cyclase (sGCα1) (9). It is integral to the NO-sGC-cGMP signaling pathway, which regulates vascular tone, smooth muscle cell relaxation, and endothelial homeostasis (10). Notably, loss-of-function mutations in *GUCY1A3* have been established as a causal factor for coronary artery stenosis, as demonstrated by Erdmann et al. (11), who linked impaired nitric oxide signaling due to *GUCY1A3* deficiency to the development of vascular stenosis by impairing nitric oxide signaling, thereby promoting vascular stenosis. In the context of MMD, Hervé et al. (12) were the first to report the co-segregation of *GUCY1A3* gene mutations with disease phenotypes through genome-wide linkage analysis and exome sequencing in MMD families. Subsequent studies further confirmed *GUCY1A3* as a key susceptibility gene for moyamoya disease (13). However, genome-wide association studies (GWAS) have yet to map *GUCY1A3* as a susceptibility locus for MMD (14).

To resolve this discrepancy and directly test the causal relationship between *GUCY1A3* loss-of-function and MMD vascular pathology, we generated a *Gucy1a3* loss-of-function mouse model. We integrated histopathological, imaging, and immunohistochemical analyses to evaluate arterial wall morphology, intimal-medial thickness, and collateral vessel formation. We also assessed whether *Gucy1a3* loss-of-function mice exhibits vasoconstrictive remodeling, characterized by external diameter narrowing of the internal carotid artery (ICA) and middle cerebral artery (MCA) preceding internal diameter stenosis—in short, a reduction in arterial external diameter. The current study aimed to systematically characterize the vascular phenotype of *Gucy1a3* loss-of-function mice, determining whether they recapitulate pathological and radiographic features of MMD to validate its potential as a preclinical model for this condition.

## 2 Experimental procedures

### 2.1 Generation and identification of *Gucy1a3*^−/−^

*Gucy1a3* loss-of-function mice (*Gucy1a3*^−/−^) were generated as described previously (15). Briefly, the loss of function of *GUCY1A3* was simulated by deleting the 13-bp base of exon 5 using the CRISPR/Cas9 gene editing system. Homozygous mutants (*Gucy1a3*^−/−^) were obtained by heterozygous crossbreeding. The model was confirmed by Sanger sequencing and semiquantitative analysis at the protein level. Sanger sequencing showed a 13-bp deletion in exon 5 of *Gucy1a3* in the homozygous mutant mice. The Western blot analysis revealed near-complete absence of α1-type soluble guanylate cyclase (sGCα1, encoded by the *GUCY1A3* gene) protein expression in brain tissue from *Gucy1a3*^−/−^ (as shown in Supplementary Figure 1).

### 2.2 Experimental animals

Male C57BL/6J wild-type mice (Wt) and *Gucy1a3*^−/−^ (8–12 weeks, 23–27 g) were used. All animal experiments were conducted in compliance with protocols approved by the Animal Care and Use Committee of Guangxi Medical University and followed the guidelines of the Chinese National Institutes of Health for laboratory animal welfare. Mice were housed under specific pathogen-free (SPF) conditions at the Animal Experiment Center of Guangxi Medical University, with controlled temperature (22–25 °C), a 12-h light/dark cycle, and free access to food and water.

### 2.3 Magnetic resonance angiography of intracranial arteries in mice

Experiments were performed using a dedicated small-animal scanner with a 5-cm bore operating at 7.0 Tesla field strength (Bruker, Ettlingen, Germany). A high-resolution three-dimensional gradient-echo time-of-flight sequence was used to acquire magnetic resonance angiography (MRA) images. The parameter details are similar to those previously described (16, 17). Cerebrovascular images were reconstructed using Radiant DICOM Viewer software (version 2023.1, Medixant, Poznan, Poland) through multiplanar reconstruction (MPR) and maximum intensity projection (MIP) to generate three-dimensional anatomical images of the arterial vasculature (18). Measurements of internal diameters of the end of the internal carotid artery (ICA), the proximal middle cerebral artery (MCA), and the end of the basilar artery (BA) were performed independently by two radiologists. The ratios of internal diameters (ICA/BA and MCA/BA) were subsequently derived from the mean values of measurements for statistical analysis (19, 20).

### 2.4 Blue latex and carbon black gelatin perfusion for cerebral vessel casting

Transcardiac perfusion with carbon black-gelatin solution or blue latex solutions was performed to evaluate the vascular anatomy of the leptomeningeal anastomosis and circle of Willis (21, 22). Under deep anesthesia induced by intraperitoneal administration of tribromoethanol (2,2,2-tribromoethanol, Solarbio, Cat# B2910, China) at a dose of 0.1 mL/10 g, mice underwent thoracotomy for cardiac exposure. Blood was flushed through left ventricular perfusion with physiological saline, followed by infusion of blue latex solution (60% natural latex:20% ammonium hydroxide: blue pigment = 20:20:1) or carbon black-gelatin solution (India ink:10% gelatin solution = 1:1). Perfusion success was confirmed by uniform filling of the cerebral arterial tree. Post-perfusion, mice were maintained at 4 °C for 30 min to solidify the perfusate, after which brains were harvested and fixed in 4% paraformaldehyde (Solarbio, Cat# P1110, China) for 48 h.

The vascular anatomical structures of the surface of the brain and the circle of Willis were systematically observed under a stereomicroscope (M205FA, Leica, Germany), with images captured for documentation. Two investigators independently measured the outer diameters of vascular images of the base of the brain using Fiji ImageJ software (version 1.54p, National Institutes of Health). Included were the blue latex dye-filled ICA ends, MCA proximal ends, and BA ends. The vascular diameter ratios (ICA/BA and MCA/BA) were derived from the mean values of measurements for subsequent statistical analysis. We performed vascular skeletonization and percentage area calculated the percentage area of comparable meningeal vascular regions in two groups of mice. The study of meningeal blood vessels, including total junctions and vascular branches, was performed using the skeletonization function in Fiji ImageJ software.

### 2.5 Histopathological analysis of large intracranial arteries

The brains of mice were carefully removed and immersed in 4% paraformaldehyde for 48 h after anesthesia. Under the guidance of a stereomicroscope, brain coronal segmentation was performed according to the trend of MCA, ICA, and BA to obtain brain tissue blocks containing corresponding blood vessels. These brain tissue blocks were paraffin-embedded separately after the standard dehydration protocol. Serial sections 4 μm thick were obtained at 30 μm intervals using a paraffin slicer for histopathological analysis (23).

The sections were stained with hematoxylin and eosin (H&E) and elastic van Gieson (EVG) staining (Solarbio, Cat. # G1597, China). For smooth muscle cell visualization, sections were immunostained using a primary antibody against alpha-smooth muscle actin (α-SMA) primary antibody (Proteintech, Cat. # 14395-1-AP, China). Antigen retrieval utilized 0.01 M sodium citrate buffer (pH 6.0) with heat mediation. After 5% goat serum blocking and primary antibody incubation, the specimens were incubated with horseradish peroxidase-conjugated secondary antibody (Proteintech, Cat. # PK1006, China). The area of positive staining was detected using a chromogenic solution with 3,3-diaminobenzidine (DAB). All sections were imaged under a fluorescence microscope (BX53, Olympus, Japan) equipped with a digital camera. Internal and external diameters of ICA, MCA, and BA segments were measured from H&E-stained pictures using Fiji ImageJ software. For *α*-SMA immunohistochemically stained images, the thickness of the tunica media at similar locations in the vessel was measured multiple times to assess vascular remodeling (24). Two independent investigators performed blinded measurements, with final values representing the mean of duplicate determinations.

### 2.6 Quantification of cortical microvascular density and diameter

To investigate cerebral cortex microangiogenesis, we performed immunohistochemical staining of coronal brain sections at the level of the hippocampus using anti-CD31 antibody (Servicebio, Cat. # GB11063-2, China), followed by DAB staining as previously described. Images were acquired using an EVOS M7000 pathology slide scanner (Thermo Fisher Scientific, United States). Appropriate exposure time, gain, and contrast settings ensured optimal image quality. Three similar cortical regions of the unilateral cerebral hemisphere were randomly selected for each mouse brain section. Microvessel density, area, and diameter were independently quantified by two blinded researchers using Fiji ImageJ software.

### 2.7 Statistical analysis

Statistical analyses were performed via GraphPad Prism (version 9.5.1, La Jolla, United States). All values are shown as the mean ± standard deviation (SD). Comparisons between the two groups were performed via the non-parametric Mann–Whitney *U* test. Values of *p* < 0.05 were considered significant.

## 3 Results

### 3.1 Intracranial arterial evaluated by MRA in *Gucy1a3*^−/−^

**Figure 1:**
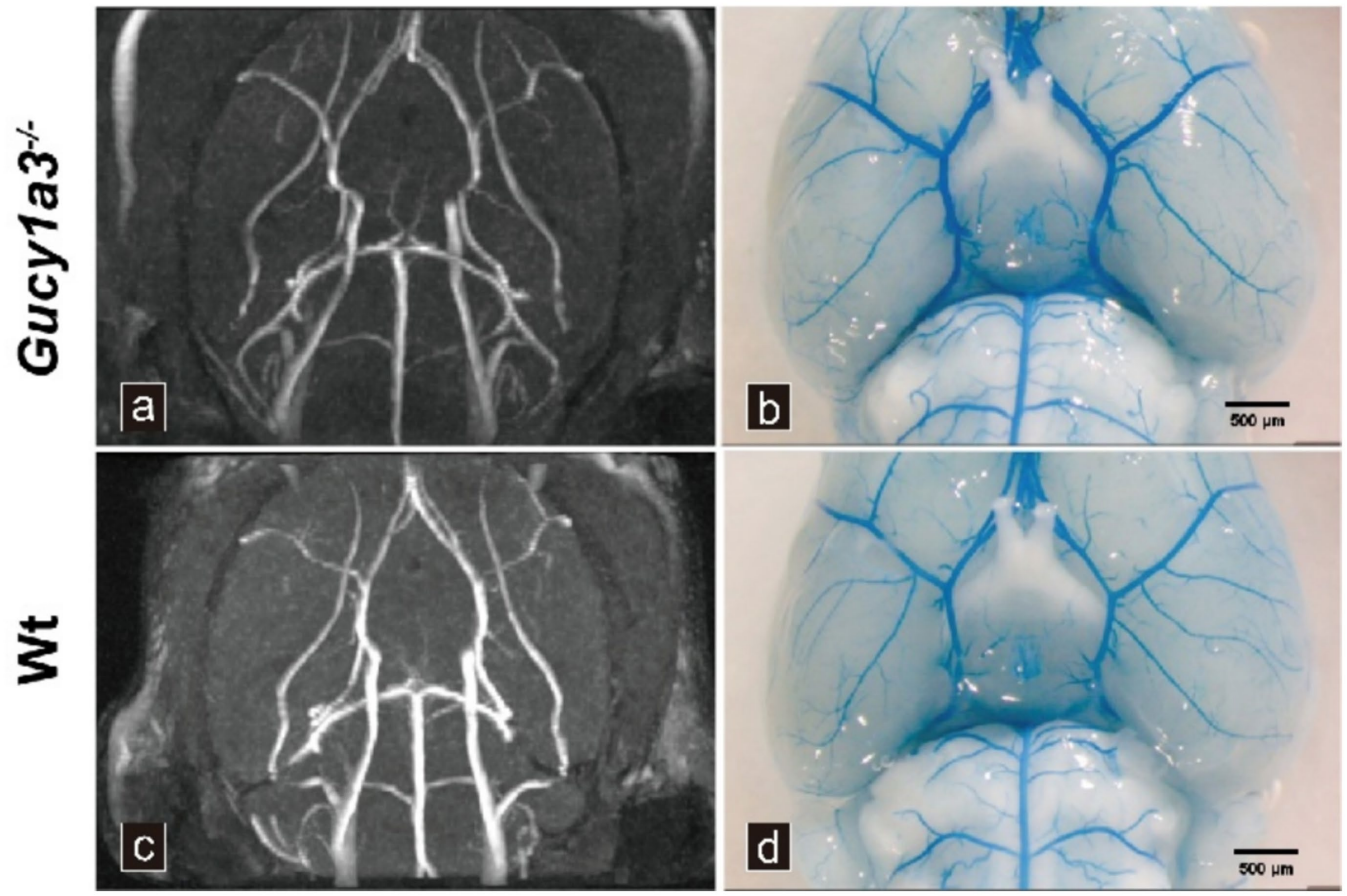
Cerebrovascular phenotype analysis of *Gucy1a3*^−/−^ and Wt via MRA and perfusion techniques. **(a,c)** 7.0-T MRA images of intracranial arteries (*n* = 7 mice per group). **(b,d)** Blue latex perfusion revealed circle of Willis anatomy (*n* = 7 mice per group). Scale bar: 500 μm.

MRA revealed similar vascular structures in 8–12-week-old *Gucy1a3*^−/−^ and Wt at this stage, with no significant short-term differences in intracranial arterial morphology within the current observation window. There were no stenotic/occlusive changes in the internal carotid arteries and their branches bilaterally. In addition, the moyamoya neovascular network, which is characteristic of human MMD pathology, was not detected at the base of the skull in either group of mice (Figures 1a,c).

### 3.2 Absence of MMD-associated large vascular features in *Gucy1a3*^−/−^

Transcardiac perfusion with blue latex solution demonstrated preserved vascular architecture of the circle of Willis in 8–12-week-old *Gucy1a3*^−/−^, compared with Wt, with no structural differences detected during the observation period. No stenotic or occlusive changes were observed in the terminal ICA and proximal MCA, and no moyamoya-like abnormal vascular networks formed at the base of the brain (Figures 1b,d).

### 3.3 Disruption of meningeal microvascular angiogenesis in *Gucy1a3*^−/−^

**Figure 2:**
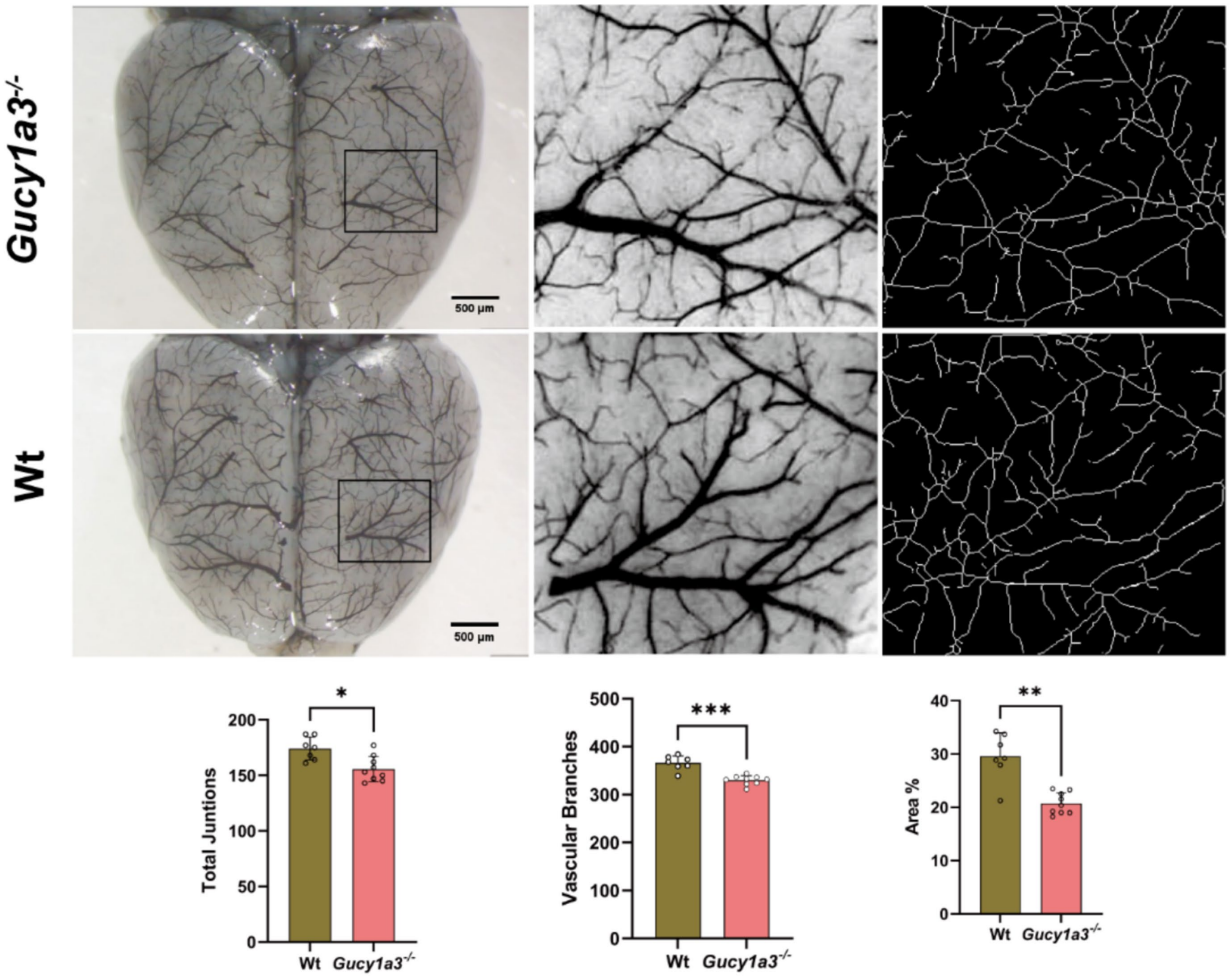
Carbon black-gelatin solution perfusion demonstrates cerebral surface vasculature (*n* = 9 mice per group). The total number of vascular junctions (174.10 ± 10.43 versus 155.80 ± 11.39, *p* = 0.0101), vascular branches (365.90 ± 14.92 versus 330.00 ± 9.72, *p* = 0.0003), and vascular area ratio (Vascular area %, 29.57 ± 4.40 versus 20.75 ± 2.01, *p* = 0.0021) in the soft meninges of *Gucy1a3*^−/−^ were significantly less than those in Wt. ^***^p < 0.001, ^**^p < 0.01, and ^*^p < 0.05. Scale bar: 500 m.

Carbon black gelatin was used for casting the meningeal vasculature. At 8–12 weeks of age, the vascular structure of the soft meninges in *Gucy1a3*^−/−^ showed no significant differences compared to that of Wt of the same age. Neither group exhibited marked hyperemia (capillary dilation). Skeletonized vascular networks were analyzed using Fiji ImageJ software, revealing a significant difference in the density of soft meningeal septa between the two groups (Figure 2).

### 3.4 No significant changes in the structure of the large intracranial arteries in *Gucy1a3*^−/−^

**Figure 3:**
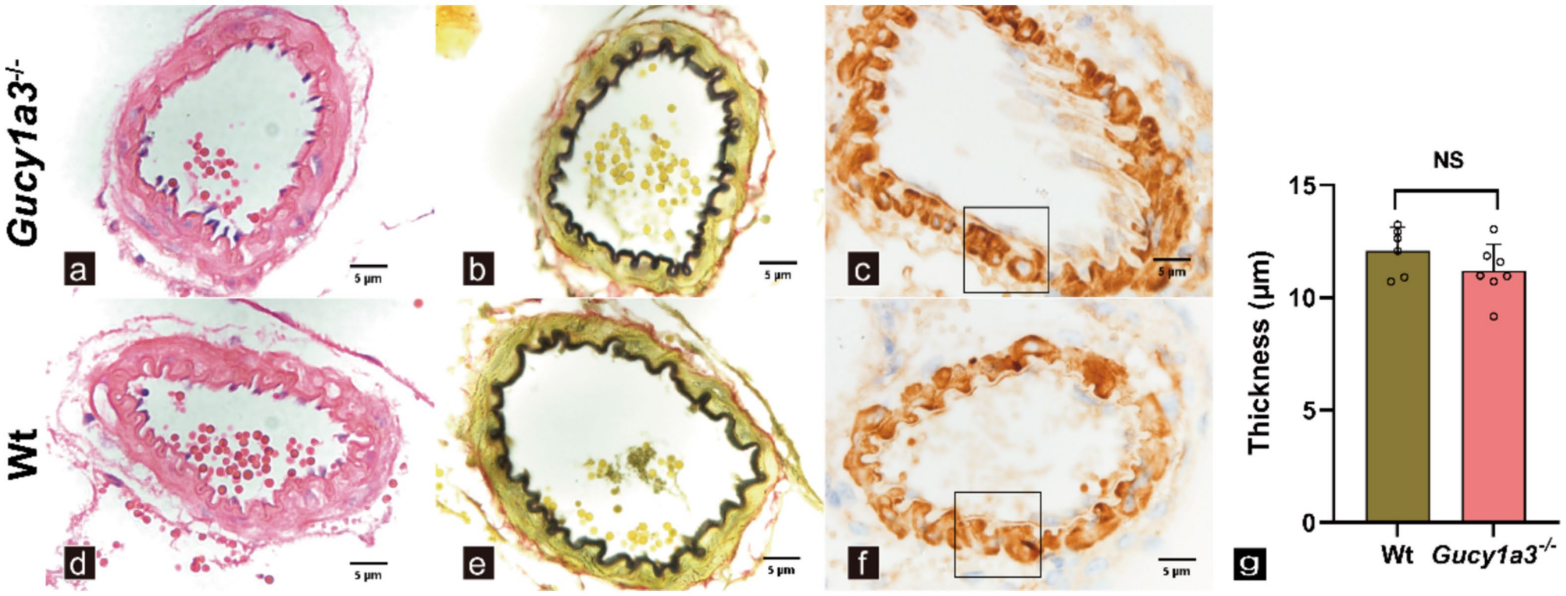
Histopathological analysis of cerebral vasculature in *Gucy1a3*^−/−^ and Wt. **(a,d)** Hematoxylin–eosin staining of the intimal layer (*n* = 8 mice per group). **(b,e)** Elastic van Gieson staining of the internal elastic lamina (*n* = 8 mice per group). **(c,f)** Immunohistochemical staining for alpha-smooth muscle actin (*n* = 7 mice per group). **(g)** Quantification of the thickness of the vascular tunica media. No significant structural differences were observed among the intima, elastic fibers, and tunica media (12.09 ± 1.07 versus 11.19 ± 1.19, *p* =0.3660) between *Gucy1a3*^−/−^ and Wt. **(a–f)** Shows high-magnification images under a 100× objective field of view. The rectangular area indicates the region of interest. NS, no significant. Scale bar: 5 μm.

MMD is characterized by hyperplastic vascular remodeling, including tunica intima thickening and tunica media thinning of intracranial arteries (24, 25). Histopathological evaluation demonstrated preserved cerebrovascular architecture in 8–12-week-old *Gucy1a3*^−/−^ compared to Wt. H&E staining revealed no evidence of MMD-characteristic intimal hyperplasia, with tunica intima composed of 1–2 endothelial cell layers in both groups (Figures 3a,d). EVG staining confirmed structural integrity of the internal elastic lamina in *Gucy1a3*^−/−^, with no elastic fiber fragmentation, duplication, or delamination observed (Figures 3b,e). Subsequent immunohistochemical analysis of *α*-SMA demonstrated intact tunica media architecture, with no degradation or loss of smooth muscle cells in cerebral arteries (Figures 3c,f). Quantitative analysis revealed no significant difference in tunica media thickness between *Gucy1a3*^−/−^ and Wt during the 8–12-week observation period (Figure 3g).

### 3.5 No vasoconstrictive remodeling in *Gucy1a3*^−/−^

We measured intracranial arterial internal diameters using MRA, determined external diameters through blue latex-perfused casting specimens, and acquired both parameters via H&E staining in 8–12-week-old mice. At this sample size and under the current experimental window, *Gucy1a3*^−/−^ exhibited no statistically significant differences in the internal/external diameters, ICA/BA and MCA/BA ratios compared to Wt (*p* > 0.05). These findings indicate no evidence of vasoconstrictive remodeling—an extremely early manifestation of MMD—was observed in *Gucy1a3*^−/−^(see Table 1).

**Table 1:** ICA/BA and MCA/BA ratios of vascular diameter on MRA, blue latex perfusion, and histopathology.

Item	Ratio	Wt	*Gucy1a3* ^−/−^	*p*-value
MRA	ICA/BA ratio (%)	92.4 ± 7.75	91.55 ± 6.68	0.60
	MCA/BA ratio (%)	69.13 ± 9.11	68.84 ± 13.12	0.97
Blue latex perfusion	ICA/BA ratio (%)	85.35 ± 2.42	86.16 ± 2.88	0.65
	MCA/BA ratio (%)	73.85 ± 5.10	69.27 ± 7.28	0.41
Histopathology (outer diameter measure)	ICA/BA ratio (%)	87.03 ± 8.03	86.66 ± 10.52	0.96
	MCA/BA ratio (%)	75.03 ± 8.50	78.39 ± 9.19	0.46
Histopathology	ICA/BA ratio (%)	57.79 ± 13.54	48.20 ± 6.18	0.23
(Inner diameter measure)	MCA/BA ratio (%)	63.29 ± 14.46	58.06 ± 7.60	0.61

Values are the mean ± SD. BA, basilar artery; ICA, internal carotid artery; MCA, middle cerebral artery; MRA, magnetic resonance angiography; *Gucy1a3*^−/−^, *Gucy1a3* loss-of-function mutant mice; Wt, wild type mice.

### 3.6 Impaired cortical microvascular angiogenesis in *Gucy1a3*^−/−^

**Figure 4:**
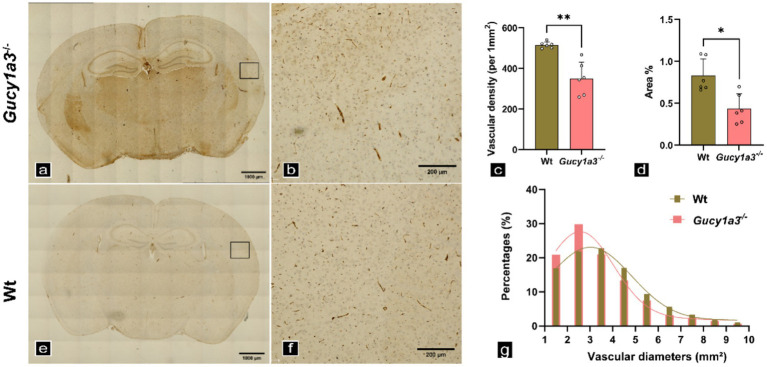
Quantification of cortical microvascular density and diameter in *Gucy1a3*^−/−^ and Wt. **(a,e)** Low-magnification (10×) CD31 immunohistochemical staining of cerebral cortex microvasculature. **(b,f)** High-magnification (20×) views of cortical microvessels. **(c)** Quantification of microvascular density. **(d)** Quantification of microvascular area. **(g)** Distribution of microvascular diameters. The cortical microvascular density and vascular internal diameters of *Gucy1a3*^−/−^ were significantly decreased compared to Wt (Vascular density, 515.70 ± 15.53 versus 351.20 ± 80.69, *p* = 0.0022; Vascular areas %, 0.83 ± 0.20 versus 0.44 ± 0.18, *p* = 0.0152). Sample size: *n* = 6 mice per group. The rectangular area indicates the region of interest. ^**^p < 0.01, ^*^p < 0.05 compared to Wt. Scale bar: 1,000 μm **(a,e)** and 200 μm **(b,f)**.

In addition to the meningeal vessels, this study further examined whether 8–12-week-old *Gucy1a3*^−/−^ develop MMD-like pathologies in cerebral perforating vessels. The quantitative analysis of vascular density and area in the stained images (Figures 4a,b,e,f) revealed that, compared with wild-type (Wt) mice, *Gucy1a3*^−/−^ exhibited significantly reduced microvascular density and area in the cerebral cortex (*p* < 0.01, *p* < 0.05) (Figures 4c,d). Subsequent analysis of vascular luminal diameters revealed that the majority (approximately 85%) of small vessels in the mutant cortex exhibited reduced diameters relative to Wt cerebral microvessels (Figure 4g).

## 4 Discussion

In this study, we systematically evaluated whether *Gucy1a3*^−/−^ could spontaneously develop MMD vascular phenotypes using multimodal approaches. Our results demonstrated that *Gucy1a3*^−/−^ developed normally under normal physiological conditions, with no significant structural differences in major cerebral arteries, the circle of Willis, or large vessel wall architecture (including the intima, internal elastic lamina, and media) compared to Wt. Histopathological analyses further revealed that the intracranial large vessel walls of *Gucy1a3*^−/−^—including the tunica media, internal elastic lamina, and tunica intima—were structurally indistinguishable from those of Wt. Quantitative analysis confirmed the absence of early MMD phenotypes, such as arterial stenosis or constrictive remodeling. Collectively, these findings suggest that *GUCY1A3* loss-of-function is insufficient to independently instigate the large vessel pathology characteristic of MMD.

Interestingly, our analysis of the leptomeningeal vasculature revealed that *Gucy1a3*^−/−^ exhibited a significant reduction in vascular branching and density. This finding appears to contradict the “congestion” or capillary ectasia often described in advanced MMD. We propose several explanations for this discrepancy. First, our loss-of-function model was studied under normoxic conditions without underlying large artery stenosis. In the absence of chronic hypoxic or hemodynamic stress—a key trigger for compensatory angiogenesis in MMD—the observed reduction in vascularity may represent a baseline defect in angiogenic capacity or vascular maintenance, rather than a failed compensatory response. This interpretation is supported by our prior work demonstrating that *GUCY1A3* deficiency impairs angiogenesis *in vivo* and *in vitro*, associated with downregulated *VEGFA/HIF-1α* signaling and diminished endothelial cell proliferation and migration (15). This suggests that the compromised angiogenic machinery in *Gucy1a3*^−/−^ may preclude a robust compensatory response, potentially explaining why the characteristic hypoxic-driven capillary “ectasia” of advanced MMD was not observed under the normoxic conditions of our study. Second, MMD vascular changes are dynamic and stage-dependent. The “congestion/dilation” phenotype, often highlighted in clinical imaging, typically emerges not as an initial lesion but specifically as an adaptive response to chronic cerebral hypoperfusion. In this context, our data likely capture an earlier or pre-symptomatic vascular phenotype, before the onset of significant hemodynamic stress that would trigger such compensatory remodeling. This interpretation aligns with clinical observations (11–13, 26) demonstrating that *GUCY1A3*-related MMD cases predominantly manifest during early childhood (6/7 cases) and are frequently associated with ischemic manifestations and endothelial progenitor cell (EPC) dysfunction, a profile suggesting an underlying vascular vulnerability that precedes overt stenosis. This suggests a potential model for studying the “vascular insufficiency” precursor state that precedes overt moyamoya collaterals. Third, different susceptibility genes may drive distinct vascular phenotypes (14). The mechanisms by which *RNF213* and *GUCY1A3* variants influence vascular remodeling may differ, leading to varied pathological outcomes depending on genetic context and experimental conditions.

The microvascular phenotype observed in *Gucy1a3*^−/−^—reduced cortical vascular density and diameter—bears resemblance to alterations reported in *Rnf213* loss-of-function models (27). While the microvascular reduction in *Gucy1a3*^−/−^ appears to be a primary defect under physiological conditions, changes in *Rnf213*^−/−^ models may reflect impaired compensatory responses to hemodynamic challenge. Nonetheless, both models highlight the importance of genetic regulation in microvascular integrity and suggest that distinct susceptibility genes may converge on shared pathways contributing to microvascular insufficiency.

The NO-sGC-cGMP signaling pathway plays a pivotal role in vascular smooth muscle relaxation, tone regulation, and angiogenesis. While reduced sGC activity has been linked to vascular anomalies (12), MMD pathogenesis is likely driven by polygenic synergy and environmental factors. Large vessels exhibit high structural stability (28), and compensatory mechanisms mediated by genes such as *RNF213* or *ACTA2*, or pathways like *VEGF* and *Notch*, may preserve vascular integrity despite *GUCY1A3* deficiency. For instance, the *Notch* pathway and *VEGFA* signaling synergistically regulate arterial/venous differentiation and embryonic vascular development (28, 29). Moreover, studies (11) have shown that *Gucy1a3*^−/−^ develop thrombotic tendencies only under specific conditions (e.g., localized trauma). The microvascular “rarefaction” in *Gucy1a3*^−/−^ likely stems from a primary deficit in the *NO-sGC-cGMP* pathway, crucial for endothelial health and angiogenesis. This deficit may create a compromised vascular bed where compensatory signals from pathways like *VEGF* or *ACTA2* are insufficient, or where concomitant dysfunction in intersecting pathways (e.g., *RNF213*’s role in endothelial metabolism and stability) synergistically drives pathology.

Beyond cell-autonomous vascular defects, recent single-cell studies highlight dynamic immune-vascular crosstalk in cerebrovascular and neuroinflammatory contexts (30). *GUCY1A3* deficiency, by impairing *NO-cGMP* signaling—a pathway known to modulate leukocyte adhesion and endothelial activation—may create a perivascular milieu conducive to low-grade inflammation. This could potentially prime the vasculature for subsequent pathological remodeling upon secondary insults. Future investigation into immune cell profiles and neuroinflammatory markers in *Gucy1a3*^−/−^ brains would be valuable to explore this interplay.

Notably, parallels exist between *GUCY1A3* and *RNF213* in MMD pathogenesis. Although *RNF213 p.R4810K* is a major susceptibility variant for MMD (31), neither knockout (32) nor knock-in (19) animal models develop spontaneous MMD phenotypes, even after extended observation periods (up to 180 weeks). Intriguingly, *Rnf213*^−/−^ mice exhibit pathological angiogenesis in the cortex, hippocampus, and retina (33), while zebrafish models show aberrant cephalic vascular sprouting (31). These findings suggest a dual mechanism for *RNF213* in MMD: gain-of-function effects promoting angiogenesis and loss-of-function effects reducing vascular stability (34). The distinct contributions of *GUCY1A3* and *RNF213* highlight the need to investigate their interactions and synergistic effects in disease progression.

This study has several limitations. First, the observation window was restricted to 8–12 weeks, which may not capture later-onset, age-dependent vascular phenotypes analogous to the adult-onset peak in human MMD. This is particularly relevant considering murine models of other MMD-associated genes, such as *Rnf213* knockout mice, which occasionally require extended rearing periods (e.g., up to 60–80 weeks). Future studies should extend this period to 80 weeks or longer. Second, although GWAS have not strongly implicated *GUCY1A3* in MMD, this may reflect limited power to detect rare variant contributions or synergistic effects—as evidenced by reported synergism between *NF1* and *MRVI1* or between *GUCY1A3* and *CCT7* in large artery atherosclerosis (35). Further studies are needed to evaluate whether rare variants in *GUCY1A3*, alone or in combination with other genes, contribute to MMD risk. To model the polygenic nature of MMD, future work should prioritize generating and characterizing double- or multi-gene mutant mice. A critical step is crossing *Gucy1a3*^−/−^ with carriers of the East Asian founder variant *Rnf213 p.R4810K* (or a complete *Rnf213* knockout), as these two genes represent major susceptibility loci. Additional crosses with models of other implicated genes (e.g., *Acta2* for smooth muscle dysfunction) could reveal synergistic or modifier effects. Third, environmental factors such as chronic ischemia-hypoxia, radiation (36), or inflammatory (37) stimuli were not incorporated. Given their documented roles in MMD, such interventions could better model the disease’s multifactorial nature. For example, subjecting mutants to chronic cerebral hypoperfusion (e.g., unilateral carotid stenosis) or mild inflammatory stimuli to unmask latent phenotypes. Fourthly, a deep molecular characterization is essential to move from phenotype to mechanism. We propose a multi-omics approach on freshly isolated cerebral microvessels or vascular single-cell suspensions from *Gucy1a3*^−/−^ and control mice. Integrating transcriptomic or proteomic analyses—for example, examining markers of endothelial dysfunction, hypoxia response (e.g., *HIF-1α, VEGFA*), and vascular instability as seen in other models (38)—would provide crucial mechanistic insights into the molecular consequences of *GUCY1A3* loss and identify potential therapeutic targets. Finally, strategies aiming to boost cGMP signaling (e.g., via sGC stimulators) or to protect endothelial function might be particularly relevant for MMD patients with *GUCY1A3* variants, potentially in combination with interventions targeting other susceptibility pathways.

## 5 Conclusion

In summary, *GUCY1A3* loss-of-function leads to cortical and meningeal microvascular “rarefaction” but does not recapitulate the large vessel pathology typical of MMD. Our findings indicate that *GUCY1A3* plays a crucial role in maintaining microvascular homeostasis and in developmental angiogenesis, with its loss necessitating complementary genetic or environmental factors to precipitate large vessel lesions. Long-term longitudinal monitoring of aging *Gucy1a3*^−/−^ is warranted to determine whether macrovascular pathology emerges over time. Simultaneously, it is necessary to elucidate the interactions between *GUCY1A3* and other MMD-associated genes (such as *RNF213*) with environmental triggers, as well as the underlying molecular mechanisms. Such insights will advance our understanding of MMD pathogenesis and inform targeted therapeutic strategies.

## Data Availability

The raw data supporting the conclusions of this article will be made available by the authors, without undue reservation.
