# Correction: Cerebrovascular phenotype analysis in *Gucy1a3* loss-of-function mice: insights into moyamoya disease susceptibility

**DOI:** 10.3389/fneur.2026.1880700

**Published:** 2026-06-16

**Authors:** Pingkai Wang, Tian Wang, Jipeng Yu, Jianli Li, Xiaozuo Lin, Yinan Zeng, Xiaohan Zhang, Shanghua Su, Man Luo

**Affiliations:** Department of Neurology, First Affiliated Hospital of Guangxi Medical University, Nanning, China

**Keywords:** cerebrovascular phenotype, *Gucy1a3* loss-of-function mice, magnetic resonance angiography (MRA), moyamoya disease, vasoconstrictive remodeling

In the published article, author “Pingkai Wang” was erroneously assigned to affiliation “Department of Neurology, Western Theater General Hospital of Chinese People's Liberation Army, Chengdu, China”. This affiliation has now been removed for author “Pingkai Wang”.

Affiliation “First Affiliated Hospital of Guangxi Medical University, Nanning, China” was omitted for author “Pingkai Wang” and this has now been added for the author.

Authors “Pingkai Wang, Tian Wang, Jipeng Yu” was erroneously omitted as equal contributing author. The corrected version appears below:

Pingkai Wang^†^, Tian Wang^†^, Jipeng Yu^†^, Jianli Li, Xiaozuo Lin, Yinan Zeng, Xiaohan Zhang, Shanghua Su and Man Luo^*^

Department of Neurology, First Affiliated Hospital of Guangxi Medical University, Nanning, China

^†^These authors have contributed equally to this work

There was a mistake in Figure 4 as published. In Figure 4, panel g, the unit on the x-axis title was incorrectly written with a superscript square “mm^2^”. The correct unit should be “mm” without the superscript “2”, squared.

The corrected Figure 4 appears below:

**Figure 4:**
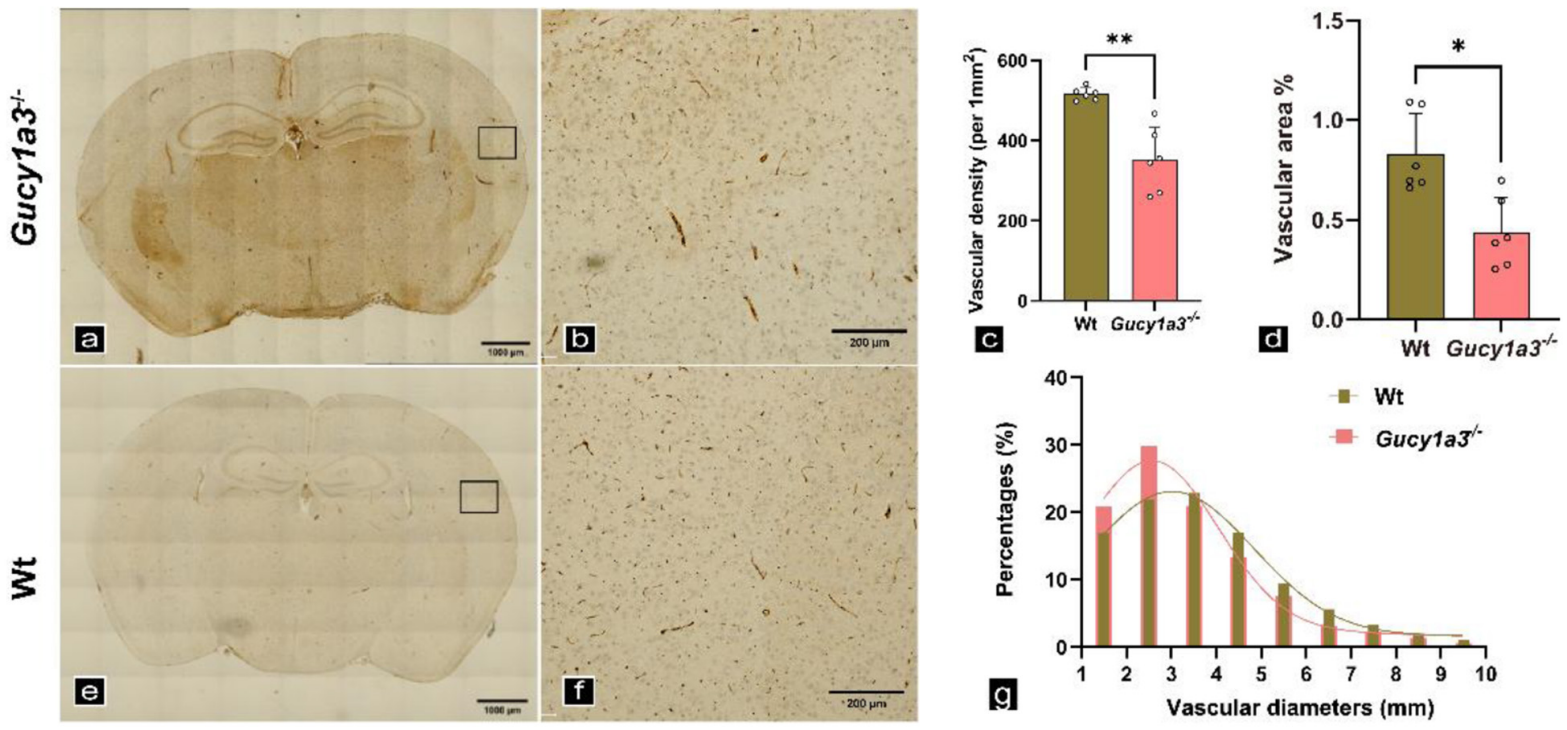
Quantification of cortical microvascular density and diameter in *Gucy*1*a*3^−/−^ and Wt. **(a, e)** Low-magnification (10×) CD31 immunohistochemical staining of cerebral cortex microvasculature. **(b, f)** High-magnification (20×) views of cortical microvessels. **(c)** Quantification of microvascular density. **(d)** Quantification of microvascular area. **(g)** Distribution of microvascular diameters. The cortical microvascular density and vascular internal diameters of *Gucy*1*a*3^−/−^ were significantly decreased compared to Wt (Vascular density, 515.70 ± 15.53 vs. 351.20 ± 80.69, *p* = 0.0022; Vascular areas %, 0.83 ± 0.20 vs. 0.44 ± 0.18, *p* = 0.0152). Sample size: *n* = 6 mice per group. The rectangular area indicates the region of interest. ^**^p < 0.01, ^*^p < 0.05 compared to Wt. Scale bar: 1,000 μm **(a, e)** and 200 μm **(b, f)**.

The original article has been corrected.

