# Supplementary figures and images for: Cerebrovascular phenotype analysis in Gucy1a3 loss-of-function mice: insights into moyamoya disease susceptibility
Source: Front Neurol. 2026 Apr 13;17:1669177. doi: 10.3389/fneur.2026.1669177 (PMC13111162; doi:10.3389/fneur.2026.1669177)

**Supplementary Figure 1**


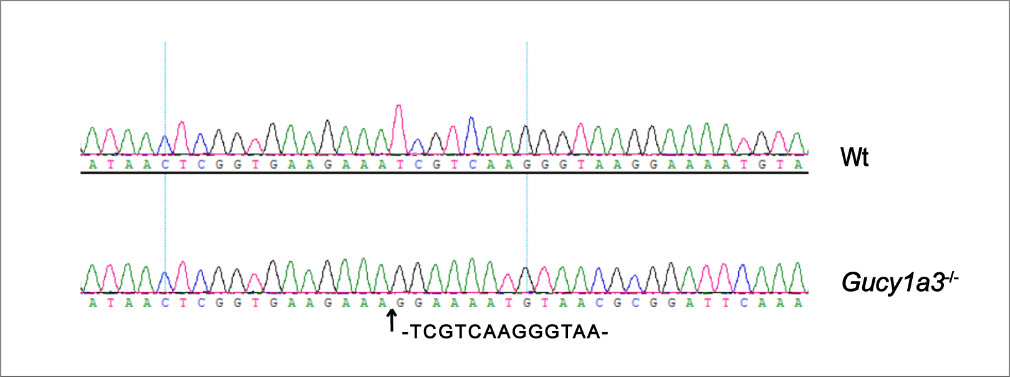

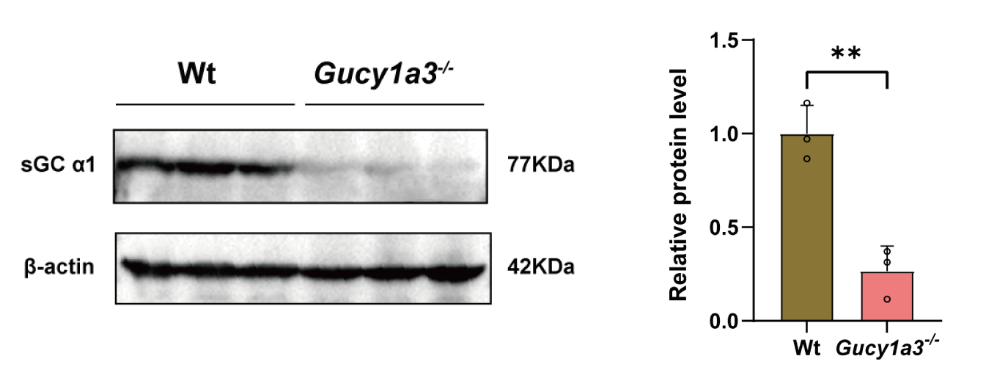

Supplement: Supplementary file 2 [file Supplementary_file_1.docx]
